# Community Structures of Phytoplankton with Emphasis on Toxic Cyanobacteria in an Ohio Inland Lake during Bloom Season

**DOI:** 10.4236/jwarp.2017.911083

**Published:** 2017-10-31

**Authors:** Ke Chen, Joel Allen, Jingrang Lu

**Affiliations:** 1 Southwest University of Science and Technology, Mianyang, China; 2 US EPA ORD, Cincinnati, OH, USA

**Keywords:** Phytoplankton, Cyanobacteria Bloom, Toxin Producer, Lake

## Abstract

The community structures of phytoplankton are important factors and indicators of lake water quality. Harmful algal blooms severely impact water supply, recreational activities and wildlife habitat. This study aimed to examine the phytoplankton composition and variations using microscopy, and identify harmful Cyanobacteria in weekly samples taken from four sites at Harsha Lake in southwest Ohio. Over the course of the summer in 2015, the phytoplankton of Harsha Lake consisted mainly of 13 taxa belonging to Bacillariophyta, Chlorophyta, Cryptophyta, Cyanobacteria, Dinophyta and Euglenophyta. Their significant successions started with Bacillariophyta and/or Chlorophyta, then bloomed with Cyanobacteria and ended with Chlorophyta and/or Dinophyta. Cyanobacteria members: *Microcystis*, *Planktothrix*, *Dolichospermum*, *Aphanizomenon*, *Cylindrospermopsis*, and *Oscillatoria* from the *Cyanophyceae* were identified to be dominant genera. These organisms varied spatially and temporally in similar patterns along with the variations of nutrients and formed the summer bloom with the total biomasses ranging from 0.01 to 114.89 mg L^−1^ with mean of 22.88 mg L^−1^. *M. aeruginosa* and *P. rubescens* were revealed as the microcystin producers, while *A. circinalis* and *Aphanizomenon* sp. were identified as a saxitoxin producer through cloning and sequencing PCR products of *mcyA*, *mcyE* and *sxtA* genes. The biomasses of phytoplankton, Cyanobacteria and *Microcystis* were positively correlated to nutrients, especially to total nitrogen. The total ELISA measurement for microcystin positively correlated with Cyanobacteria (R^2^ = 0.66, P < 0.0001), *Microcystis* (R^2^ = 0.64, P < 0.0001) and phytoplankton (R^2^ = 0.59, P < 0.0001). The basic information on the occurrence and biomasses of Cyanobacteria and total phytoplankton, and the analysis for toxic species, which were the first report for the inland water in Ohio, USA, will document the succession patterns of phytoplankton and toxin production over a season and provide data to predict risk occurrence to both human and ecological factors.

## 1. Introduction

Investigation of phytoplankton by direct microscopy yields information of biomass and species compositions, which has long been used in lake studies [1], such information is often of considerable significance in the monitoring of lake water quality [2]. Toxic algal blooms in inland water are mostly caused by the excessive growth of cyanobacteria [3]. Those harmful algal blooms (HABs) can damage freshwater ecosystems, cause human health problem due to release of cyanotoxin and odors to source water and kill fish and shellfish [4]. Many HAB species present a risk to the health of humans or animals and other animals by their toxins and other bioactive compounds [5]. Over the past 10 years, HABs have been increasing in Ohio inland lake waters [6]. Cyanotoxins including microcystin and saxitoxin were detected from 74% of the samples collected from Ohio source and recreational surface waters [7]. The concentrations of microcystin were higher than the Ohio Recreational Public Health Advisory 3 level of 6 μg L^−1^ in 44% samples and were higher than 20 μg L^−1^ (part of the Recreational No Contact Advisory 4) in 31% of samples [7]. William H. Harsha Lake or Harsha Lake, a multi-use reservoir and primary drinking water source in southwest OH, has experienced an increase in HAB frequency and intensity over the past several decades. Previously, there have been extensive investigations on the chemical contaminants and nutrients of this lake, such as denitrification [8], total organic carbon concentrations [9] and pesticides [10]. Recently, HABs and associated toxins are a major water-quality issue not only in Lake Erie, but also in the other inland lakes in Ohio. Therefore, monitoring of HABs in this lake has also been carried out [6] [7]. However, the pattern of phytoplanktonic community successions and toxin producing species during blooms has not been documented. Phytoplankton are an important basal resource to heterotrophic organisms in lakes, and their growth, succession and community structures determine the potential productivity of the ecosystem [11] as well as the status of the ecosystem and water quality. There are increasing concerns about the impacts on water quality, especially visual appearance, tastes and odors associated with Cyanobacteria blooms on the lake [12]. Phytoplankton are often considered to be indicators of water quality [13]. For example, the community composition and succession, as assessed by alterations of the species and their abundances over time, can indicate changes in the physical and/or chemical status of the water [13]. The study on the phytoplankton community, especially toxic cyanobacteria, in a typical Ohio inland lake will provide a basis for further investigation of biomasses and variations of toxic cyanobacteria and provide an important reference for other Ohio inland lakes. Thus the purpose of this investigation was to examine phytoplankton composition with emphasis on Cyanobacteria, community succession and HAB toxic species and to explore the relationships between the variations of phytoplankton and HABs, and biomasses and nutrients.

## 2. Materials and Methods

### 2.1. Study Area

Harsha Lake is an open reservoir, located 25 miles east of Cincinnati in southwestern Ohio (Latitude: 39.0132285, Longitude: −84.1148988). The lake, which was built in 1978 [10] and is used for flood control, water supply, recreation, and as a wildlife habitat [8] [14], is estimated to have generated more than thirty million US dollars in visitor expenditures and to have prevented more than seventy million US dollars in flood damages since its impoundment [9]. The mean depth of Harsha Lake is 13.1 m with the maximum water depth of 34 m and a water depth more than 8 m in most areas [15]. It covers an area of 8.7 km^2^ and drains from a watershed of 890 km^2^ with 64% of land use for agriculture and 26% comprised of forest cover. The lake also serves as the surface water source for the Bob McEwen Drinking Water Treatment Plant (10 millions of gallons per day) (Personal communications).

### 2.2. Sampling

**Figure 1:**
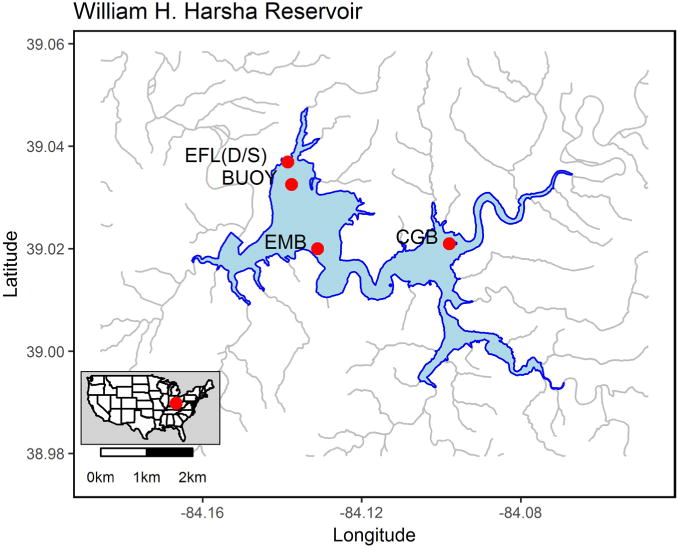
Harsha Lake map and sampling sites.

Samples were collected on Harsha Lake for the biological, physico-chemical and cyanotoxin analysis. Weekly (from May to October, 2015) samples were collected using a plastic water jug, which was rinsed using 5% hydrochloric acid and deionized water, to scoop up water from the surface (~0.5 m depth). There were four sites including east fork lake at drinking water treatment plant intake (EFLS: latitude 39.0367, longitude −84.1381), Harsha Buoy (BUOY: latitude 39.032506, longitude −84.137661), camp ground beach (CGB: latitude 39.022506, longitude −84.094618) and east fork lake main beach west of narrows (EMB: latitude 39.02, longitude −84.1311) (Figure 1). One-liter water samples, which were put into autoclaved sample bottles, were collected and 100 - 200 mL aliquots were filtered using EMD Millipore Durapore™ membrane filter (0.40 μm, MilliPore, Foster City, CA) for DNA extraction. For phytoplankton, 200 mL raw water was preserved in 1% Lugol’s solution buffered with acetic acid and the phytoplankton was concentrated by sedimentation for 24 h followed by removal of all but 25 mL of the water using a 5 mL disposable serological pipette. The final volumes for identification and enumeration varied from 5 to 10 mL depending on observed densities.

### 2.3. Identification and Enumeration of Phytoplankton

Phytoplankton were identified to genus level under a 400× magnification using an Nikon microscope (Nikon Corp., Japan) following taxonomic instructions [16]. To enumerate phytoplankton cells, two slides and a minimum of 20 fields for each slide were examined using a hemacytometer (Hausser Scientific, Reichert-Jung, Horsham, PA). To estimate biomass, mean linear dimensions of individual unit (algal cells and filament fragments) were measured for more than twenty individuals according to geometric shapes described by Sun and Liu [17]. The measurements were calibrated using a standard scale bar (S22-StageMic; Graticules Ltd., UK) mounted on the microscopic objective for the microscopic ocular. After information on taxa and linear dimensions were input into a Microsoft Excel worksheet, the biovolume of the algal cells and filament fragments were calculated using the equations for various shapes simulated by Sun and Liu (2013).

### 2.4. DNA Extraction, PCR, Cloning and Sequencing

DNA extractions were conducted using AllPrep DNA (QIAGEN, Valencia, CA) following manufacturer’s instruction. To examine the toxin producers or those potential toxic genera found by microscopy, PCR analysis targeting conserved sequences of cyanotoxin biosynthesis and genus-specific genes were done. The targeted genes included *mcyA* (CD1 assay) and *mcyE* (HEP assay) for the producers of various genera (Table 1) of microcystin, cylindrospermopsin biosynthesis genes (*cyr*) and RNA polymerase gene (*rpoC*) for toxic and total *Cylindrospermopsis*, saxitoxin (*sxtA*) for toxic *Dolichospermum* and *Aphanizomenon*, and nodularin biosynthesis gene (*nda*) and total populations (*nts*) for *Nostoc* (Table 1). The 25 μl PCR mixtures contained 2 μl of template DNA, a 0.2 mM concentration of each of the four deoxynucleoside triphosphates (dTTP, dCTP, dGTP, and dATP), 1.5 mM MgCl2, 1 μM (each) primer, and 2.5 U of TaqDNA polymerase (Clone Tech, Mountain View, CA). Each PCR for specific assay was conducted with total of twelve DNA extracts isolated from EFLS and BUOY samples collected during June to September. Thermocycling conditions included 1 min of denaturation at 94°C, 1 min of primer annealing at the specific temperature (°C) indicated in Table 1, and 5 min of primer extension at 72°C. This cycle was repeated 25 times. When a sample was positive, the amplicons containing genus-specific sequences were cloned using a pCR4.1 TOPO *E. coli* kit (Invitrogen, Carlsbad, CA) to aid in further identification. Individual clones (8 colonies for each library) were sequenced by using BigDye Terminator chemistry (Life Technology), in order to confirm the targets. Raw sequences were edited using Sequencher (Gene Codes Corp., Ann Arbor, MI). Phylogenetic trees were constructed from the alignments of sequences based on the neighbor joining method. The software mega v6 [18] was used to build trees using 1000 replicates to develop bootstrap confidence values. Representative *mcyA*, *mcyE*, *rpoC* and *sxtA* gene sequences from clone libraries were deposited in GenBank with accession numbers: KY117603-KY117652.

### 2.5. Analysis of Microcystin and Other Parameters

Measurements of microcystin (MC) were measured in raw and filtered water using the MC-ADDA Enzyme Linked Immunosorbent Assay (ELISA) kit (Abraxis, Warminster, PA), which quantifies the β-amino acid ADDA (all-S all-E)-3-Amino-9-methoxy-2,6,8-trimethyl-10-phenyldeca-4,6-dienoic acid. The mean percent recovery for each laboratory fortified sample matrix and duplicate set should be greater than, or equal to, 60% and less than, or equal to, 140% of the true value. Both raw water and filters underwent three freeze-thawing cycles prior to proceeding measurement procedures introduced by the ELISA manufacturer. Surface water temperatures were taken during sampling. Nutrients [total nitrate (TNO3), total nitrite (TNO2), total nitrogen (TN), total ammonia (TNH4), soluble reactive phosphorus (SRP), total phosphorous (TP), Table 2] are measured using the Latchat Quickchem 8000, Flow Injection Analysis, Autoanalyzer (Hach Co, USA) according to manufacturer’s instruction.

### 2.6. Data Analysis

Pearson correlations were calculated between factors (nutrients, temperatures and MC) and phytoplanktonic and cyanobacterial biomasses. The multiple comparisons of between months and between locations were also conducted to examine the evenness and variations of phytoplanktonic distribution.

## 3. Results

### 3.1. Biomass and Composition of Phytoplankton Community

**Figure 2:**
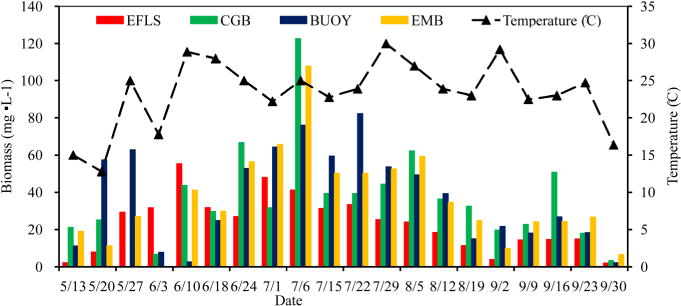
Weekly distributions of total phytoplankton biomass at the four sites.

**Figure 3:**
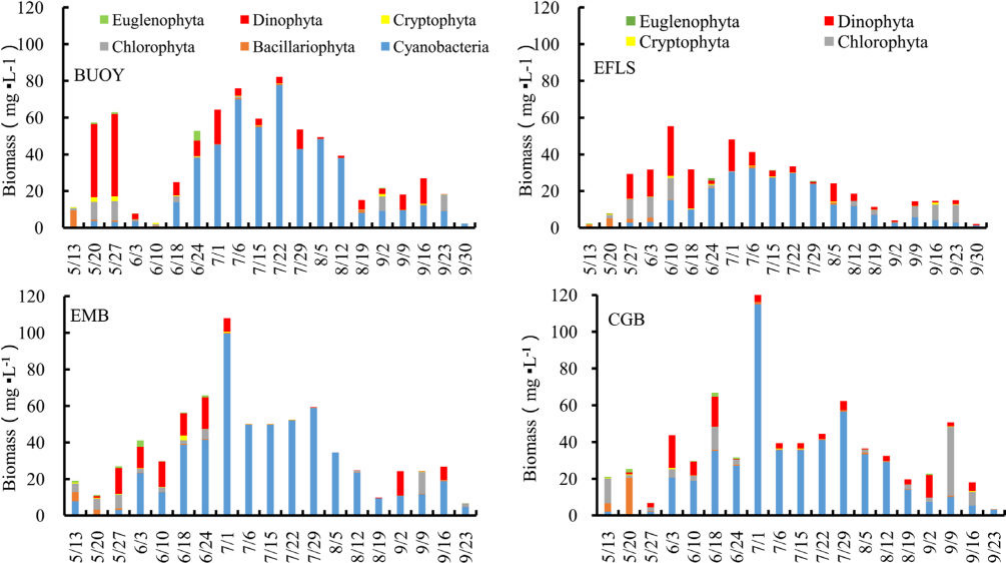
Weekly relative biomass abundance of the total phytoplankton community of the six major phyla found in Harsha Lake from May to September.

During the entire sampling period, the phytoplankton of Harsha Lake consisted mainly of 13 genera belonging to Bacillariophyta (*Cyclotella*, *Melosira* and *Synedra*), Chlorophyta (*Chlamydomonas* and *Pediastrum*), Cryptophyta (*Cryptomonas*), Cyanobacteria (*Aphanizomenon*, *Dolichospermum*, *Microcystis* and *Oscillatoria*), Dinophyta (*Ceratium* and *Peridinium*) and Euglenophyta (*Euglena*), and their total biomass varied from 2.06 to 122.72 mg L^−1^ with a mean of 34.12 mg L^−1^ (Table 3). There were significant variations over time at 0.05 level (Figure 2 and Table 4). The biomasses were lower during May and September and had a peak at each site in June or July. For example, peaked at CGB (122.72 mg L^−1^) and EMB (107.94 mg L^−1^) on July 6^th^, at BUOY (82.27 mg L^−1^) on July 22^nd^ and at EFLS (55.33 mg L^−1^) on June 10^th^. The composition of phytoplankton at the phylum level was dominated by Cyanobacteria (67%), following with Dinophyta (18.63%), Chlorophyta (9.45%), Bacillariophyta (2.94%), Cryptophyta (1.00%) and Euglenophyta (0.92%). There were population successions in all the surface waters (Figure 3). For example, Bacillariophyta and Chlorophyta were the two dominant phyla, which comprised up to 90% of the total phytoplankton biomasses at the four surface water sites in early and/or mid-May. Subsequently, Bacillariophyta was replaced by Dinophyta as the dominant phyla from late May through early and/or mid-June. Thereafter, cyanobacterial biomasses reached the range of 35% to 95% and became the dominant phyla from late June through September. Cyanobacterial biomasses, which ranged from 0.01 to 114.89 mg L^−1^ with mean of 22.88 mg L^−1^, steadily increased from May to late June, remained at constant high levels from early to late July, and then slowly decreased from August through November (Figure 3).

**Figure 4:**
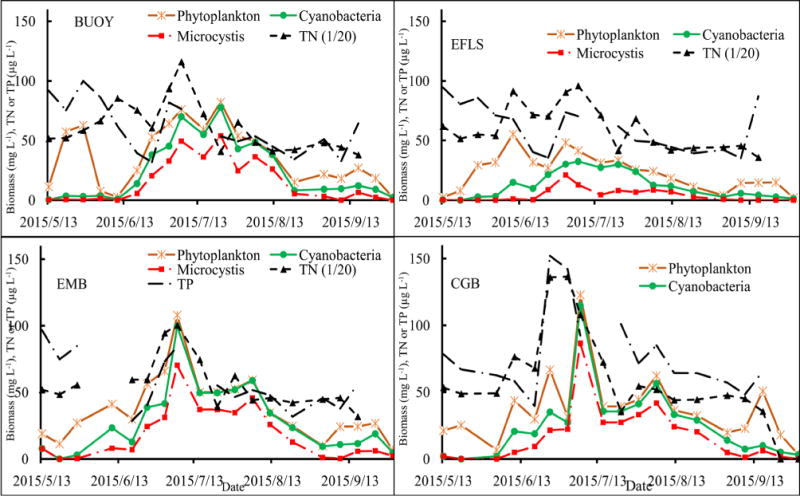
Associations of the biomass of phytoplankton, cyanobacteria and *Microcystis* with total nitrogen (TN) and total phosphorus (TP).

Among the cyanobacterial communities, *Microcystis*, *Dolichospermum* and other filamentous Cyanobacteria presented in Harsha Lake across the entire sampling period and they were dominant in the surface water from June to September. For overall surface samples, during bloom (June 24^th^ to August 19^th^), the order of relative abundance was as follows: *Microcystis* (60%) > *Dolichospermum* and other filamentous group (40%). There were significant community successions: the filamentous group dominated in late May to early June and September, while the *Microcystis* played a major role during bloom from late June through early July. The peak biomass of *Microcystis* (21.10 mg L^−1^) at site EFLS in July 1^st^ was only almost half, one third or one quarter of the biomass at sites BUOY, EMB and CGB, respectively (Figure 4).

### 3.2. Associations of the Biomass with Nutrients and Microcystin

**Figure 5:**
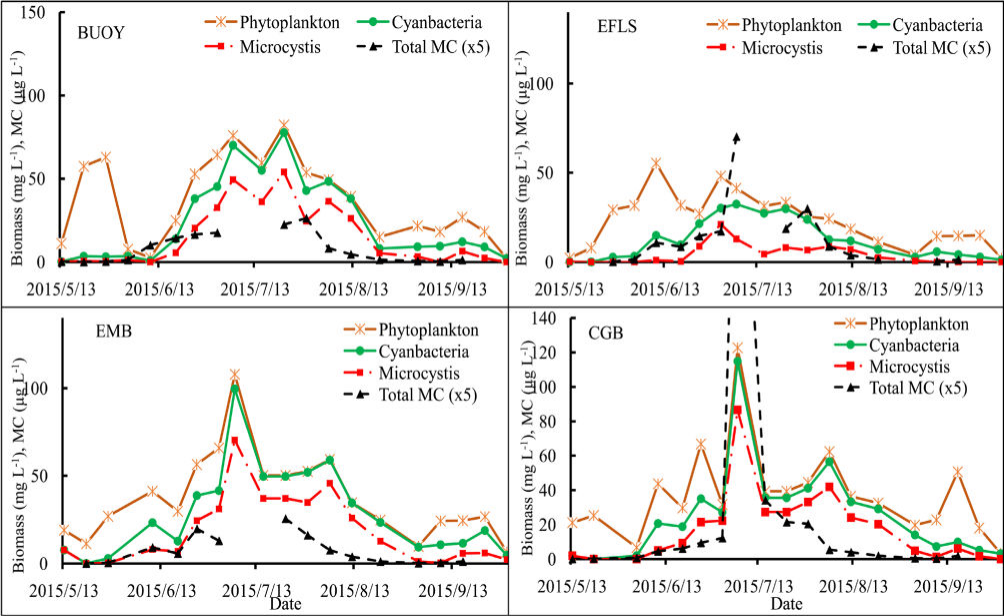
Associations of the biomass of phytoplankton, cyanobacteria and *Microcystis* with total MC measured using ELISA.

The general trends of the biomass of phytoplankton, Cyanobacteria and *Microcystis* are positively correlated to nutrients, especially to TN and TNH4 with P(R^2^) < 0.05 (Table 5, Figure 4). There were similar variation patterns of TN, TNH4 and TP in the four sites and along the variations of the biomasses. The levels of TN and TNH4 reached to two peaks in mid-June and early July, respectively (Table 2), of which the first peaks co-occurred with the phytoplankton biomasses in CGB, EFLS and EMB, while the second peaks were in agreement with the peaks of the biomasses of the three categories in the four sites. The biomasses of the three categories decreased along with the lower levels of nutrient after the second peaks. It seemed that the variations of TP and the biomasses levels were more associated after mid-June (Figure 4). The total ELISA measurement for microcystin positively correlated with Cyanobacteria (R^2^ = 0.66, P < 0.0001), *Microcystis* (R^2^ = 0.64, P < 0.0001) and phytoplankton (R^2^ = 0.59, P < 0.0001) (Table 5), indicating that majority of the microcystin were produced by *Microcystis* and there were some other MC-producers co-occurred in Harsha Lake (Figure 5).

### 3.3. Toxic Species Identified Using PCR and Sanger Chemistry Sequencing

**Figure 6:**
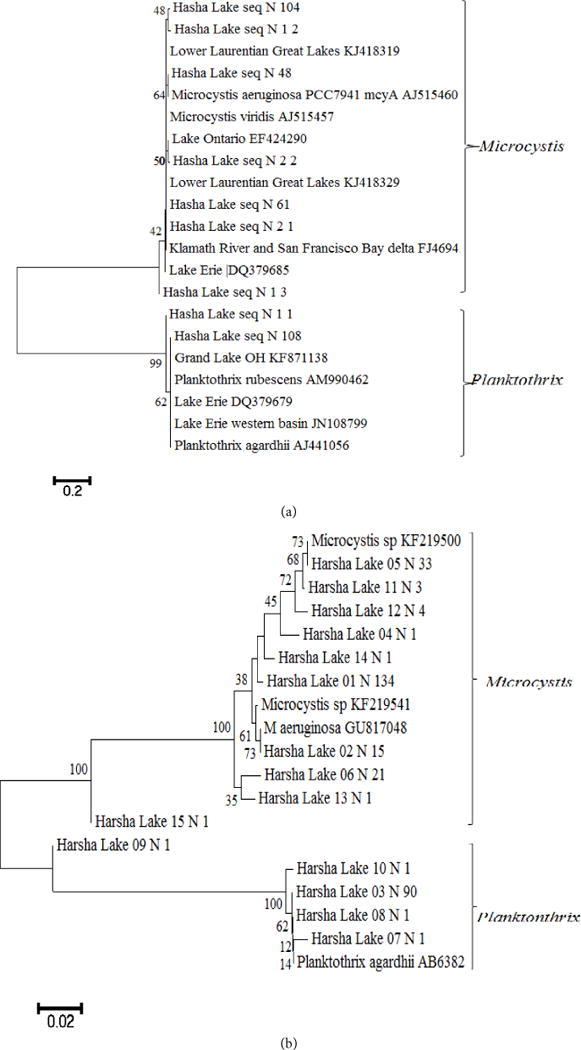
Unrooted neighbor-joining tree of *mcyA* (a) and *mcyE* (b) gene amplified for microcystin producer sequences obtained from clone libraries of the Harsha Lake water. Sequences were aligned, and bootstrap consensus trees (1000 replicate) were created with MEGA6 (1% divergence). Bootstrap values are shown at the nodes.

**Figure 7:**
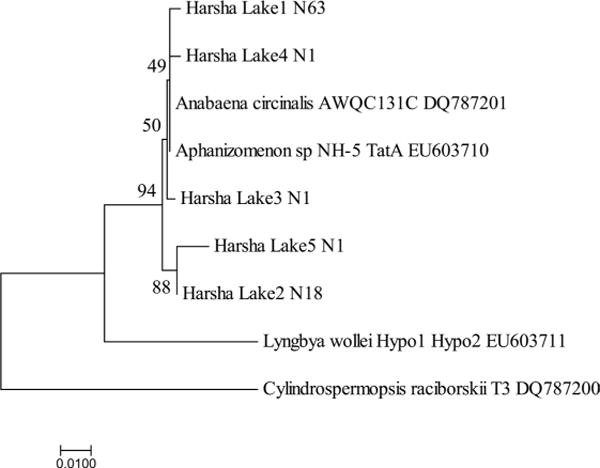
Unrooted neighbor-joining tree of *sxtA* gene amplified for *Dolichospermum* sequences obtained from clone libraries of the Harsha Lake water. Sequences were aligned, and a bootstrap consensus trees (1000 replicate) were created with MEGA6 (1% divergence). Bootstrap values are shown at the nodes.

**Figure 8:**
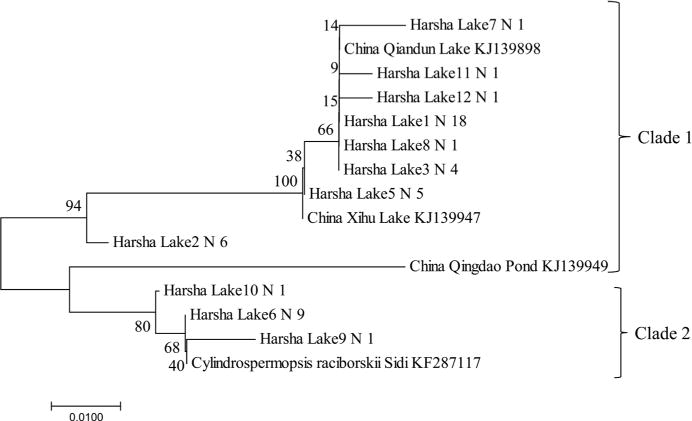
Unrooted neighbor-joining tree of *rpoB* gene amplified for *Cylindrospermopsis raciborskii* sequences obtained from clone libraries of the Harsha Lake water. Sequences were aligned, and a bootstrap consensus trees (1000 replicate) were created with MEGA6 (1% divergence). Bootstrap values are shown at the nodes.

As shown from the microscopy data, Cyanobacteria taxa that are known to potentially form HAB were observed. To confirm their presence and to further identify toxin producers of microcystin, cylindrospermopsin, saxitoxins and nodularins, the sequences of both *mcyA* (CD1) and *mcyE* (HEP) were analyzed. Two genera (*Microcystis* and *Planktothrix*) were identified and successfully distinguished based on the sequence similarity to reference sequences with an agreement of phylogenetic clusters. The two groups of *mcyA* sequences (119 for *Microcystis* with 97% consensus, and 109 for *Planktothrix* with 99% consensus) were 99% similarity to *M. aeruginosa* and 100% to *P. rubescens*, respectively (Figure 6(a)). Similarly, a total of 312 *mcyE* sequences, which were similar to *M. aeruginosa* (99%, n = 210) and *P. rubescens* (99%, n = 89) (Figure 6(b)), were also generated. The results indicated that the two major populations were potential microcystin producers and they were detected by either one of the two assays targeting to two separate genes, but the sequences using HEP assay showed more diverse due to the longer lengths and more coverages of heterogeneous sequences than those amplified using CD1 assay. Analyses were performed for the potential producers of other toxins such as cylindrospermopsin using the *cyr* (toxin) and *rpoC* (total) assays, saxitoxins using the *sxtA* assay and nodularins using the *nda* and *nts* assays, of which, the assays: *rpoC* and *nda* are the non-toxin gene targets, and their amplified sequences included both toxin or non-toxin producers. PCR amplifications for the phylogenetic analysis of the *sxtA* sequences (n = 84 sequences from 8 libraries), most sequences (99%) showed 98% identity to *Dolichospermum circinalis* and *Aphanizomenon* sp., respectively (Figure 7). For the gene sequences of the *rpoC*, *stxA* and *nts* were positive, but not for those of the *cyr* and *nda*, indicating potential producers of saxitoxin but not of the other toxins. The results confirmed the presence of non-toxic *Cylindrospermopsis* and *Nostoc.* Phylogenetic analysis on *rpoC* sequences (n = 47 sequences from 8 libraries) showed most (64%) to be 98% - 100% similar to *C. raciborskii*. All the sequences (93% similarity) could be grouped into two clades (Figure 8) with similarity to isolated sequences from various water of China, such as Xihu Lake, Qiandun Lake and Qingdao pond [19].

## 4. Discussion

The phytoplankton phyla composition observed in this study was also found in other investigated Ohio lakes such as lake Erie [20] and other smaller lakes [6] [21]. The dominance of Cyanobacteria, which was also observed in a number of Ohio lakes [6], could be typical for summer algal blooms. However, how a nontoxic-algal-bloom community shifted to a toxic algal bloom could be a key point for signaling cyanobacterial blooms, and has not been further investigated in the previous studies. The patterns of species succession at the four surface water sites were similar, starting from Bacillariophyta and/or Chlorophyta, following with Dinophyta in late spring and early summer, heavily blooming with Cyanobacteria from mid-June to mid-August, and then subsiding with the reoccurrence of Chlorophyta and/or Dinophyta. Therefore, the abundant presence of Cyanobacteria with relatively few Bacillariophyta and/or Chlorophyta was indicative to algal blooms. High occurrences of most cyanobacterial species are potentially harmful. Beaulieu *et al*. reviewed the data from 1147 lakes in the USA, and found that the bloom-forming cyanobacterial biomass average ranged from 0 to 200.26 mg L^−1^ with a mean of 1.51 mg L^−1^ [14]. In Harsha Lake, cyanobacterial species, which were mainly those dominant *Microcystis*, *Dolichospermum* and *Planktothrix*, were proved to be potentially harmful in term of the presence of toxic genes. Considering the fact that Cyanobacteria were the absolute dominant group and their biomasses ranged from 1.21 to 114.89 mg L^−1^, we concluded that HAB occurred from June to September in this Lake. Previous studies have reported that the Cyanobacteria found in this study were microcystin producers [22], so they could become a potential water-quality issue, especially for drinking water. However, in contrast with our results in 2015, previous investigations in 2013 and 2014 [6] for the same lake showed substantial differences in the community composition. During 2013, *Dolichospermum* was dominant in May, whereas in 2014 and 2015 *Dolichospermum* was present but never dominant. Likewise, during 2014 and 2015, the filamentous group of cyanobacterial was dominant in May and/or early June but did not represent a substantial portion of the cyanobacterial community in 2013. The *Microcystis*, which dominated the phytoplankton communities in the past 3 summers, showed the typical eutrophic situation for a shallow lake water [21]. As indicated from previous studies, the environmental conditions that support phytoplankton biomass, create genera dominance or trigger Cyanobacteria groups shifts may vary not only from lake to lake, or from season to season [23], but even from location to location in the same lake. Harsha Lake is a typical small water body vulnerable to HABs [24]. Our results demonstrated that surface water was well-mixed among the three sites (BUOY, CGB and EMB), because only the differences of biomasses between BUOY and EFLS, and CGB and EFLS were statistically significant using multiple comparisons. The well-mixed surface water explained that there were similar compositions and population succession patterns among the surface sampling sites (BUOY, CGB and EMB). For example, the site CGB (northeast side) had the highest biomass of phytoplankton (especially, *Microcystis*), followed by the site EMB (southwest side) and site BUOY (northwest side), while the site EFLS (northwest side) had lowest biomass of total phytoplankton and *Microcystis*. It is reasonable that the higher biomasses occurred in the beach areas (CGB and EMB), because dense algal biomass were frequently observed in the shallow water along each beach.

Generally, temperature is considered to be a key factor in triggering phytoplankton successions [25]. A number of previous studies showed that cyanobacteria adapt to grow better in higher temperature and eutrophic environments, thus in this shallow inland lake, when temperatures increased to >25°C, the Cyanobacteria succeeded from other algae and their biomass significantly increased (Figure 2). The photo-system and buoyancy regulation characteristics of *Microcystis* allows them to photo-adapt to the extreme light conditions in summer and become the absolute dominant genus in subtropical lakes [26]. For cyanobacterial dominance, nutrients played an important role. In a study on Taihu lake, Paerl *et al*. (2011) indicated that both excessive nitrogen (N) and phosphorus (P) loads might be responsible for the proliferation of non-nitrogen (N_2_)-fixing Cyanobacteria (e.g., *Microcystis*) to dominate blooms [27]. Jensen *et al.* (1994) suggested that continuous inputs of nutrients and carbon from the sediment and external sources in hypertrophic shallow lakes increased the dominance of Cyanobacteria and other algae [28]. Similar to those studied in China Taihu and Danish lake, which are hyper-eutrophic shallow lake with bloom-forming *Microcystis* and heterocystous, filamentous genera capable of N_2_ fixation (*Dolichospermum*, *Aphanizomenon*), Harsha Lake could also be heavily affected by nutrient sources of internal sediments and external watersheds. A previous analysis of cyanobacterial dominance in 143 shallow lakes along a latitudinal transect ranging from subarctic Europe to southern South America [29], indicated that the relative abundance (percent biovolume) of Cyanobacteria steeply increased with temperature and high nutrient load, suggesting there is synergistic effects of nutrients and temperatures.

Compared to other algal blooms in this lake observed by Francy (2015a), the bloom in 2015 was very stable and no abrupt decay was observed. One main reason for this may have been that there were no rapid growth bursts which in turn may be attributable to more overcast and cooler weather conditions that summer. An examination of the weather during the sampling season indicated that there were only 9 out of 20 days that were clear among the sampling dates, and only 5 clear weather days in the two-days before each sampling date. The temperatures during the sampling dates ranged from 12.8 to 30.0 with average 23.3°C, which were also much lower than those for the same durations in 2013 and 2014. Previous analyses of the effects of meteorological condition have suggested that accumulated active temperatures and sunshine duration exert important influences on cyanobacterial blooms [30]. They found that accumulated active temperature (≥18°C) above 370°C·day for three ten-day and sunshine duration more than 208 hours in Taihu Lake with eutrophication would be ready to Cyanobacterial bloom. In Harsha lake of this study, which was also a shallow lake with eutrophication, when the HAB occurred from June to September, the accumulated active temperature (≥18°C) was above 370 (°C·day ≥ 18°C) for three ten-day and sunshine duration was more than 208 hours. Other conditions like relative humidity and wind speed might have had an inconspicuous association with cyanobacterial blooms as observed by Zhang *et al*. [30].

Phylogenetic analysis in this study showed the presence of the producers of microcystin (*M. aeruginosa*, *Planktothrix*) and saxitoxin (*Dolichospermum* and *Aphanizomenon*) based on the sequences of toxin genes. Microscopic observations indicated that they were the members of the dominant genera of Cyanobacteria. The analytic data measured using ELISA showed the presence of microcystin was the majority of cyanotoxin measurements. Thus the data obtained using different approaches to reveal HAB species were consistent. In order to compare possible geographical genotypes of *M*. *aeruginosa* found between this study and others, the *mcyA* sequences retrieved from various geographical locations including Ohio and adjacent waters using CD1 assay were compared. The Harsha Lake sequences were 99-100% identical to *M. aeruginosa* and were also 99% - 100% similar to those from Lower Laurentian Great Lakes [20], Lake Erie [31], Lake Ontario [32] and even Klamath River and San Francisco Bay delta [33]. Similar to the Harsha Lake sequences that were 99% - 100% identical to *P. rubescens* Z18, sequences from Grand lake St. Marys (OH) [34] and Lake Erie [31] were also 99-100% identical to this species. The phylogenetic similarity of potentially toxic species *M. aeruginosa* among Harsha Lake and other adjacent waters, such as Lake St. Clair, Lake Erie and Lake Ontario, indicates its broad presence. It should be noticed that the fragment of *mcyA* gene may not reflect high diversity of the amplicons as observed in previous study [20], due to the little variable region of *mcyA* assay included in its short sequence (230 bp). Davis *et al*. (2014) found that there was little diversity between *mcyA* amplicons (these sequences showed genetic homogeneity) collected from each site in Lake St. Clair with all of the amplicons clustering with previously reported *M. aeruginosa mcyA* sequences. Therefore, the assay of *mcyE* was also used. Although there were still two groups (*Microcystis*, *Planktothrix*) retrieved, more diverse *Microcystis* sequences were found. Microcystins are commonly produced by Cyanobacteria in the genera *Microcystis*, *Planktothrix*, and *Dolichospermum* (*Dolichospermum*) [22]. These three genera are dominant in most Ohio lakes according to previous data [6]. Positive correlations between *Microcystis* biomass and microcystin were also observed in Great Lakes [35]. The findings of the saxitoxin (*sxt*) gene in Harsha Lake samples indicated the potential production of this neurotoxin of *A. circinalis* and *Aphanizomenon* sp. As for *Cylindrospermopsis*, the sequences observed in this study were similar to the isolate sequences from Chinese waters like Xihu Lake, Qiandun Lake and Qingdao pond, which mostly revealed no presence of toxic genes (*cyrA* and *cyrL*) [19]. However, *rpoC* based genotypes might not necessarily indicate whether *Cylindrospermopsis* showed *cyr* gene positive, considering *rpoC* gene presented in both toxic and nontoxic species, while the *cyr* gene was found to sporadically distribute in cyanobacterial strains and environmental samples [19]. Currently HAB monitoring using microscopy for phytoplankton and analytical analysis for cyanotoxin is recommended by local agencies such as OH EPA in their State of Ohio Harmful Algal Bloom Response Strategy For Recreational Waters [7]. In order to make accurate risk assessment and monitoring of cyanotoxin, molecular approaches to determine toxic species are needed. The results of this study have provided insight into the community successions, the relationships between Cyanobacteria and the other phytoplankton and presence of toxic Cyanobacteria. The continuing studies on Harsha Lake will further provide the analysis of association of Cyanobacteria blooms with environmental factors and the development of molecular monitoring tools for the toxic species.

## Figures and Tables

**Table 1:** Oligonucleotide PCR primer sequences for clone sequencing.

Target	Oligo name	Sequences (5′–3′)	Tm (°C) bp		Reference
mcyE or ndaF, for total MC producers	HEPf	TTTGGGGTTAACTTTTTTGGGCATAGTC	56	470	Jungblut and Neilan, 2006
	HEPr	AATTCTTGAGGCTGTAAATCGGGTTT			
mcyA for MC producers of *Microcystis*,	CD1f	AAAAGTGTTTTATTAGCGGCTCAT	56	302	Hisbergues, 2003
*Planktothrix* and *Dolichospermum*	CD1r	ATCCAGCAGTTGAGCAAGC			
cyrA, *Cylindrospermopsin*	cyrAf	GTCTGCCCACGTGATGTTATGAT	60	71	Al-Tebrineh *et al*., 2012
	cyrAr	CGTGACCGCCGTGACA			
*cyrL*	cyrLf	CAGGCTTATCTGCAACAACATTTCT	56	813	Jiang *et al*., 2014
	cyrLr	CGGTTTATCAGTTCCAGAGTATCCA			
rpo, *C. raciborskii*	Cy12	GGCATTCCTAGTTATATTGCCATACTA	56	234	Wilson *et al*., 2000
	Cy14	GCCCGTTTTTGTCCCTTTGCTGC			
anaC	anaC-gen F	TCTGGTATTCAGTCCCCTCTAT	58	366	Rantala-Ylinen *et al*., 2011
	anaC-gen R	CCCAATAGCCTGTCATCAA			
sxtA, *Dolichospermum*,	sxtAf	GGAGTGGATTTCAACACCAGAA	60	148	Al-Tebrineh *et al*., 2012
*Cylindrospermopsin*, *Lyngbya*	sxtAr	GTTTCCCAGACTCGTTTCAGG			
Nda, toxic*Nostoc*	ndaF	GTG ATT GAA TTT CTT GGT CG	61	188	Koskenniemi, 2007
	ndaR	GGA AAT TTC TAT GTC TGA CTC AG			
*Nts*, total *Nostoc*	*NTSf*	TGTGATGCAAATCTCA(C}A)A	56	200	Moffitt, 2001; Neilan *et al*., 1997
	1492R	TACGGCTACCTTGTTACGAC			

**Table 2:** Monthly mean values (μg L^−1^) of main nutrients in Harsha Lake.

Site	Nutrients	May	June	July	August	September
BUOY	TP	58	49	65	44	49
	TRP	39	33	45	33	34
	TN	1365	1578	1409	889	869
	TNO2	10	8	13	7	5
	TNO3	79	49	93	5	3
	TNH4	96	160	53	9	10
CGB	TP	76	79	102	69	57
	TRP	38	49	68	39	32
	TN	1067	1784	1410	911	856
	TNO2	17	16	20	7	6
	TNO3	114	307	233	7	4
	TNH4	47	160	66	11	12
EFLS	TP	90	61	65	43	55
	TRP	56	41	45	32	33
	TN	1162	1536	1472	887	837
	TNO2	19	15	16	9	7
	TNO3	266	142	130	22	22
	TNH4	88	191	88	21	32
EMB	TP	85	50	65	50	47
	TRP	51	30	43	23	37
	TN	1097	1471	1369	923	816
	TNO2	19	11	14	6	6
	TNO3	258	83	102	5	3
	TNH4	46	134	117	9	10

**Table 3:** Monthly mean biomass values (mg L^−1^) of phytoplankton in Harsha Lake.

Site	Phylum	Month				
		May	June	July	Aug	Sep
EFLS	Cyanobacteria	0.97	12.31	28.67	10.49	3.31
	Bacillariophyta	2.92	0.69	0.39	0.42	0.11
	Chlorophyta	4.26	6.30	0.14	1.78	4.80
	Cryptophyta	0.33	0.58	0.16	0.21	0.34
	Dinophyta	4.51	16.22	6.45	5.07	1.44
	Euglenophyta	0.13	0.00	0.12	0.00	0.00
BUOY	Cyanobacteria	2.33	14.02	58.18	31.53	8.40
	Bacillariophyta	3.63	0.11	0.49	0.66	0.09
	Chlorophyta	7.14	1.46	0.21	0.04	3.39
	Cryptophyta	1.89	0.38	0.18	0.03	0.45
	Dinophyta	28.23	4.72	8.10	2.48	5.03
	Euglenophyta	0.60	1.32	0.00	0.00	0.08
EMB	Cyanobacteria	3.60	24.95	58.50	38.95	11.04
	Bacillariophyta	3.09	0.27	0.33	0.20	0.26
	Chlorophyta	6.05	2.27	1.12	0.35	2.78
	Cryptophyta	0.50	1.05	0.24	0.00	0.07
	Dinophyta	5.03	12.55	4.92	0.08	4.25
	Euglenophyta	0.86	1.39	0.23	0.00	0.00
CGB	Cyanobacteria	1.02	19.14	50.89	39.61	7.94
	Bacillariophyta	12.45	0.29	0.66	0.73	0.24
	Chlorophyta	7.63	5.43	0.56	0.63	10.05
	Cryptophyta	0.41	0.27	0.15	0.04	0.19
	Dinophyta	0.54	10.97	3.09	2.73	4.38
	Euglenophyta	1.18	0.65	0.17	0.00	0.14
Average	Cyanobacteria	1.98	17.61	49.06	30.14	7.67
	Bacillariophyta	5.52	0.34	0.47	0.50	0.18
	Chlorophyta	6.27	3.87	0.51	0.70	5.25
	Cryptophyta	0.78	0.57	0.18	0.07	0.26
	Dinophyta	9.58	11.11	5.64	2.59	3.78
	Euglenophyta	0.69	0.84	0.13	0.00	0.05

**Table 4:** Monthly differences of phytoplanktonic biomass (mg L^−1^) using t tests significant at the 0.05 level indicated by ^***^.

Month comparisons	Between means	Simultaneous 95% confidence limits		Significant at the 0.05 level
8 – 7	22.02	8.17	35.86	***
6 – 7	22.10	9.15	35.05	***
5 – 7	28.19	15.47	40.90	***
9 – 7	38.79	26.80	50.78	***
6 – 8	0.08	−14.60	14.77	
5 – 8	6.17	−8.31	20.65	
9 – 8	16.77	2.93	30.62	***
5 – 6	6.09	−7.54	19.71	
9 – 6	16.69	3.74	29.64	***
9 – 5	10.61	−2.11	23.32	

**Table 5:** Correlations among the biomasses of phytoplankton, cyanobacteria and *Microcystis*, nutrients and microcystin measured using ELISA in Harsha Lake.

R^2^	rawElisa	rawMC	TN	TP	TNO2	TNH4	TNO3	Phytoplankton	*Microcystis*	Cyanobacteria
rawELISA	1	0.49215	0.0899	0.10457	0.0786	−0.0407	0.00562	0.59043	0.64101	0.66135
		<0.0001	0.1057	0.0613	0.1574	0.4651	0.9196	<0.0001	<0.0001	<0.0001
	331	331	325	321	325	325	325	124	124	124
rawMC	0.49215	1	0.00192	0.05171	−0.0811	0.05195	−0.114	0.32615	0.40439	0.45738
	<0.0001		0.9706	0.3279	0.1196	0.319	0.0283	<0.0001	<0.0001	<0.0001
	331	392	370	360	370	370	370	166	166	166
TN	0.0899	0.00192	1	0.38248	0.46201	0.60163	0.53643	0.46474	0.3707	0.40047
	0.1057	0.9706		<0.0001	<0.0001	<0.0001	<0.0001	<0.0001	<0.0001	<0.0001
	325	370	370	360	370	370	370	148	148	148
TP	0.10457	0.05171	0.38248	1	0.54635	−0.0121	0.69984	0.23572	0.11115	0.04217
	0.0613	0.3279	<0.0001		<0.0001	0.8191	<0.0001	0.0051	0.1911	0.6208
	321	360	360	360	360	360	360	140	140	140
TNO2	0.0786	−0.0811	0.46201	0.54635	1	0.16334	0.87089	0.29475	0.22141	0.17558
	0.1574	0.1196	<0.0001	<0.0001		0.0016	<0.0001	0.0003	0.0068	0.0328
	325	370	370	360	370	370	370	148	148	148
TNH4	−0.0407	0.05195	0.60163	−0.0121	0.16334	1	0.17076	0.33432	0.1891	0.2174
	0.4651	0.319	<0.0001	0.8191	0.0016		0.001	<0.0001	0.0213	0.0079
	325	370	370	360	370	370	370	148	148	148
TNO3	0.00562	−0.114	0.53643	0.69984	0.87089	0.17076	1	0.18093	0.08197	0.03881
	0.9196	0.0283	<0.0001	<0.0001	<0.0001	0.001		0.0278	0.322	0.6396
	325	370	370	360	370	370	370	148	148	148
Phytoplankton	0.59043	0.32615	0.46474	0.23572	0.29475	0.33432	0.18093	1	0.81455	0.83802
	<0.0001	<0.0001	<0.0001	0.0051	0.0003	<0.0001	0.0278		<0.0001	<0.0001
	124	166	148	140	148	148	148	166	166	166
*Microcystis*	0.64101	0.40439	0.3707	0.11115	0.22141	0.1891	0.08197	0.81455	1	0.97835
	<0.0001	<0.0001	<0.0001	0.1911	0.0068	0.0213	0.322	<0.0001		<0.0001
	124	166	148	140	148	148	148	166	166	166
Cyanobacteria	0.66135	0.45738	0.40047	0.04217	0.17558	0.2174	0.03881	0.83802	0.97835	1
	<0.0001	<0.0001	<0.0001	0.6208	0.0328	0.0079	0.6396	<0.0001	<0.0001	
	124	166	148	140	148	148	148	166	166	
